# Microbiome characterization of defensive tissues in the model anemone *Exaiptasia diaphana*

**DOI:** 10.1186/s12866-021-02211-4

**Published:** 2021-05-21

**Authors:** Justin Maire, Linda L. Blackall, Madeleine J. H. van Oppen

**Affiliations:** 1 School of Biosciences, The University of Melbourne, Melbourne, VIC Australia; 2 Australian Institute of Marine Science, Townsville, QLD Australia

**Keywords:** Symbiosis, Anemone, *Exaiptasia*, Aiptasia, Acontia, Bacteria, Metabarcoding

## Abstract

**Background:**

Coral reefs are among the most diverse and productive ecosystems on Earth. This success relies on the coral’s association with a wide range of microorganisms, including dinoflagellates of the family Symbiodiniaceae that provide coral hosts with most of their organic carbon requirements. While bacterial associates have long been overlooked, research on these microorganisms is gaining traction, and deciphering bacterial identity and function is greatly enhancing our understanding of cnidarian biology. Here, we investigated bacterial communities in defensive tissues (acontia) of the coral model, the sea anemone *Exaiptasia diaphana*. Acontia are internal filaments that are ejected upon detection of an external threat and release toxins to repel predators.

**Results:**

Using culturing techniques and 16S rRNA gene metabarcoding we identified bacterial communities associated with acontia of four Great Barrier Reef-sourced *E. diaphana* genotypes. We show that bacterial communities are similar across genotypes, and dominated by *Alteromonadaceae*, *Vibrionaceae*, *Rhodobacteraceae*, and *Saprospiraceae*. By analyzing abundant amplicon sequence variants (ASVs) from metabarcoding data from acontia and comparing these to data from whole anemones, we identified five potentially important bacterial genera of the acontia microbiome: *Vibrio, Sulfitobacter, Marivita, Alteromonas,* and *Lewinella*. The role of these bacteria within the acontia remains uninvestigated but could entail assistance in defense processes such as toxin production.

**Conclusions:**

This study provides insight into potential bacterial involvement in cnidarian defense tissues and highlights the need to study bacterial communities in individual compartments within a holobiont.

**Supplementary Information:**

The online version contains supplementary material available at 10.1186/s12866-021-02211-4.

## Background

Coral reefs provide us with countless benefits by supporting fisheries, tourism and coastal development, and their value is estimated to 9.9 trillion USD/year [1]. Additionally, they are among the most diverse ecosystems in the world and are home to more than 25% of marine species, hence making them a hallmark of biodiversity [2, 3]. Despite their immense value, coral reefs have been massively declining over the past decades as a result of anthropogenic pressures [4]. The current climate crisis is resulting in increases in frequency and intensity of summer heatwaves, which have been responsible for recurrent coral mass bleaching events in the past years and significant coral mortality [5, 6].

Corals associate with a multitude of microorganisms [7], with which they form a holobiont [8]. Of those microorganisms, endosymbiotic dinoflagellates of the Symbiodiniaceae family [9, 10] have received the most attention so far; by translocating photosynthate into coral tissues, they provide their host with most of their organic carbon needs [11–13]. As essential as the Symbiodiniaceae are, their association with corals is also extremely fragile; increases in water temperatures can result in the breakdown of this symbiosis, leading to symbiont loss, a phenomenon known as ‘coral bleaching’ [14]. Nevertheless, bacteria have also recently been recognized as crucial members of the coral holobiont [15, 16]. The flexibility of coral bacterial community composition during thermal stress events [17–19] has raised the possibility of a bacterial role in coral bleaching tolerance and overall health. Potential other roles of coral-associated bacteria include carbon, nitrogen and sulfur cycling, and host protection through the production of antimicrobial compounds or competition with opportunistic bacteria [15, 20]. Bacteria are found in all microhabitats in a coral polyp, including the mucus [21], skeleton [22], tissue layers [23, 24], gastrodermal cavity [25], the mesoglea [26], and even inside the Symbiodiniaceae cells [27]. However, only a handful of studies have compared community structure in the different body parts of the polyp [23, 28, 29], where a high variability across the different compartments was found.

**Fig. 1:**
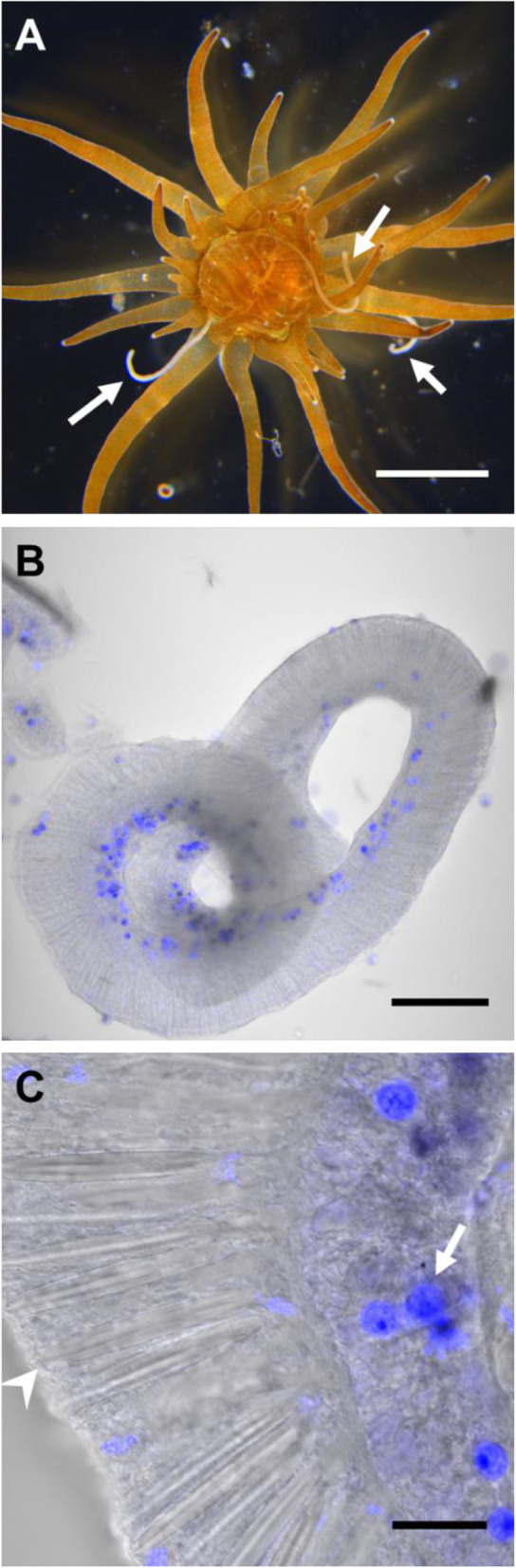
Acontia structure in *E. diaphana* (genotype AIMS1). **a**: Aboral view of an anemone observed in light microscopy ejecting several acontia (white arrows). Scale bar: 200 mm. **b-c**: Visualization of acontia (grey, transmitted light) in fluorescence microscopy highlighting the presence of Symbiodiniaceae (blue, white arrow in **c**). Also note the abundant presence of nematocysts, containing spring structures that are expelled towards predators (white arrowhead in **c**). Scale bars: 100 μm in B; 20 μm in C. Blue and grey are not the real colors

*Exaiptasia diaphana* (formerly *Exaiptasia pallida* and commonly referred to as “Aiptasia”) has been widely used as a model for cnidarian-Symbiodiniaceae interactions [30, 31], notably because of its easy laboratory maintenance, ability to reproduce sexually and asexually, and associations with similar Symbiodiniaceae to its coral relatives. Recent studies have also focused on the bacterial communities associated with different genotypes of *E. diaphana* [32–36]. However, compartmentalized analyses of bacterial communities are still lacking in this coral model. Here, we used four genotypes of Great Barrier Reef-sourced *E. diaphana* [37] and investigated the bacterial communities present in a defensive tissue, the acontia. Acontia are white coiled filaments that extend from the mesenterial filaments near the pedal disk, and contain numerous cnidocytes [38–40]. Upon detection of an external threat, acontia are ejected through cinclide pores [38, 39, 41] (Fig. 1a) and dart-like tubules discharged from nematocysts [42] (Fig. 1b-c) penetrate the predator and release toxins that may repel the predator [43, 44]. Acontia subsequently retract and recoil within the anemone [39]. Acontia have also been shown to act internally, without being ejected, by releasing nematocysts on ingested preys, hence potentially aiding prey disintegration and digestion [45]. Acontia are functionally similar to sweeper tentacles, found in most scleractinian corals, although the latter are usually used to compete with nearby corals for space [46].

Through culture-dependent and -independent techniques, we characterized the bacterial microbiome of *E. diaphana*’s acontia and isolated a wide diversity of acontia-associated bacteria. This first report of bacteria present in an anemone’s acontia suggests a possible bacterial involvement in the anemone’s defense system.

## Results

### *E. diaphana*’s acontia associate with Symbiodiniaceae and bacteria

To evaluate the presence of microorganisms in *E. diaphana*, acontia ejection was triggered by poking anemones and acontia were subsequently dissected and observed with confocal laser scanning microscopy (CSLM). Symbiodiniaceae are highly autofluorescent and were detected within the acontia by CLSM (Fig. 1b-c and Additional file 1); their abundance was found to be much lower than in tentacles (Additional file 1A).

Furthermore, we used a culture-dependent approach to assess the existence of acontia-associated bacterial communities. A total of 150 bacteria colonies were isolated from agar culture plates on which the dissected and homogenized acontia of the four anemone genotypes were spread. Each isolate was morphologically described and identified through 16S rRNA gene sequences and phylogenetic analysis (Additional files 2 and 3). Isolates spanned 17 genera within five classes: *Alphaproteobacteria*, *Gammaproteobacteria*, *Flavobacteriia*, *Actinobacteria* and *Bacilli*. Of the 150 bacterial isolates, *Alteromonas* and *Muricauda* were cultured from all genotypes. Cultured isolates belonging to *Vibrio, Thalassotalea,* and *Erythrobacter* were also common.

### 16S rRNA gene metabarcoding reveals bacterial communities of acontia are diverse, with abundant ASVs being shared among host genotypes

As culture-dependent methods tend to under-represent the diversity of bacterial communities, we moved to a culture-independent method to capture a wider bacterial diversity in acontia. We performed 16S rRNA gene metabarcoding on whole acontia dissected from anemones from all four genotypes. Sequencing produced 416,814 reads across acontia (*n* = 3 per genotype), extraction blanks (*n* = 1), and no template PCRs (*n =* 1). After merging, denoising and chimera filtering 270,326 reads remained. After removal of contaminants, 690 amplicon sequence variants (ASVs) were observed across the acontia samples.

**Fig. 2:**
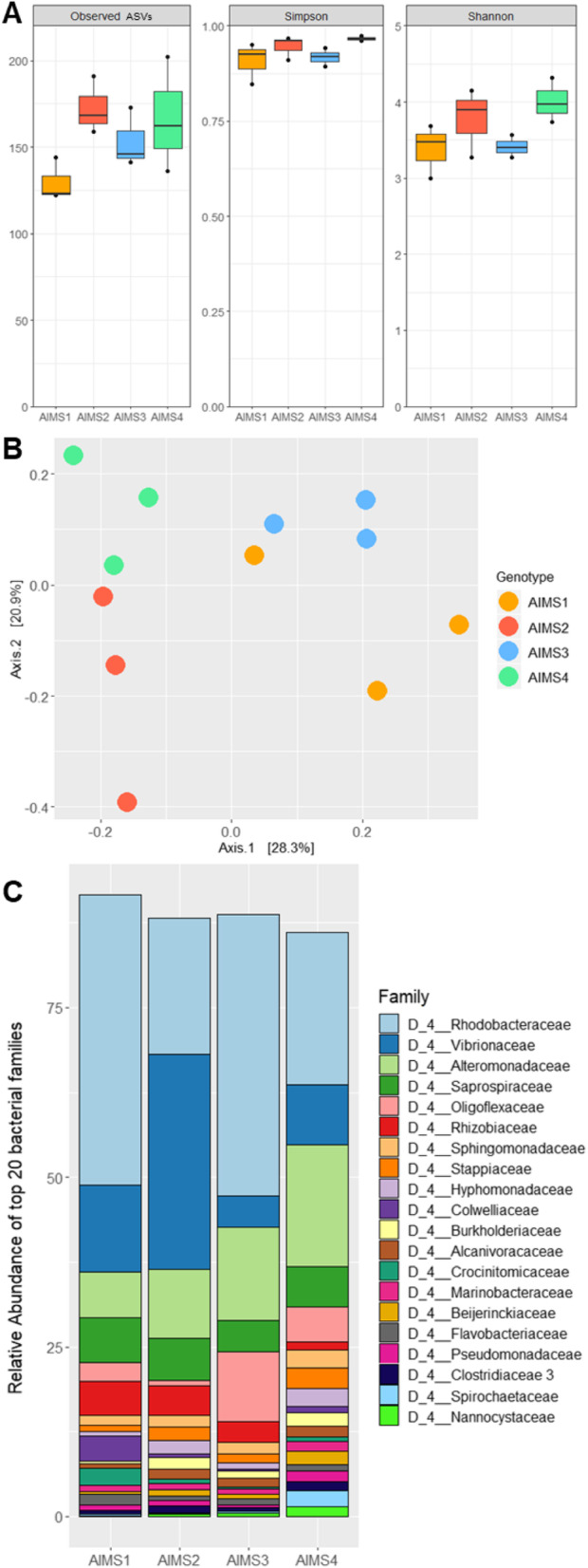
16S rRNA gene metabarcoding reveals a wide bacterial diversity in acontia of *E. diaphana*. **a**: Alpha-diversity metrics (Observed ASVs, Simpson’s Diversity Index [1-D], Shannon’s Index) of the bacterial communities present in acontia from genotypes AIMS1–4. Boxes represent the first and third quartiles for three independent replicates, the bar represents the median, and dots represent minimum and maximum values. No significant differences between genotypes were found in any of the alpha-diversity metrics based on ANOVAs. **b**: PCoA visualization of beta-diversity of the bacterial communities in acontia from genotypes AIMS1–4, based on Bray-Curtis dissimilarity matrices. Each point is an individual sample. Genotype was considered as a significant effect based on a PERMANOVA (999 permutations, *p* = 0.001), but no pairwise comparisons between genotypes was found significant (PERMANOVA, 999 permutations). **c**: Relative abundance of bacterial families in acontia from genotypes AIMS1–4. For each genotype, three independent replicates were merged

Alpha-diversity metrics (observed ASVs, Simpson’s index, Shannon’s index) showed no significant differences between genotypes based on ANOVAs (*F*_ObsASVs(3,8)_ = 0.55, *p* = 0.66; *F*_Simp(3,8)_ = 0.72, *p* = 0.57; *F*_Shan(3,8)_ = 0.41, *p* = 0.75) (Fig. 2a). Principal coordinate analysis (PCoA) visualization of beta-diversity using the Bray–Curtis dissimilarity index revealed some degree of clustering of data points by genotype (Fig. 2b), and PERMANOVA testing (999 permutations) showed that bacterial community structure varied significantly with anemone genotype (*F*_(3,8)_ = 2.42, *p* = 0.001). However, pairwise comparisons between all genotypes did not yield any significant differences, suggesting a degree of consistency between the acontia from different genotypes. Close inspection of community composition confirmed these similarities (Fig. 2c), with around 70% of all reads belonging to the four same bacterial families in all four anemone genotypes (*Alteromonadaceae*, *Vibrionaceae*, *Rhodobacteraceae*, and *Saprospiraceae*).

**Fig. 3:**
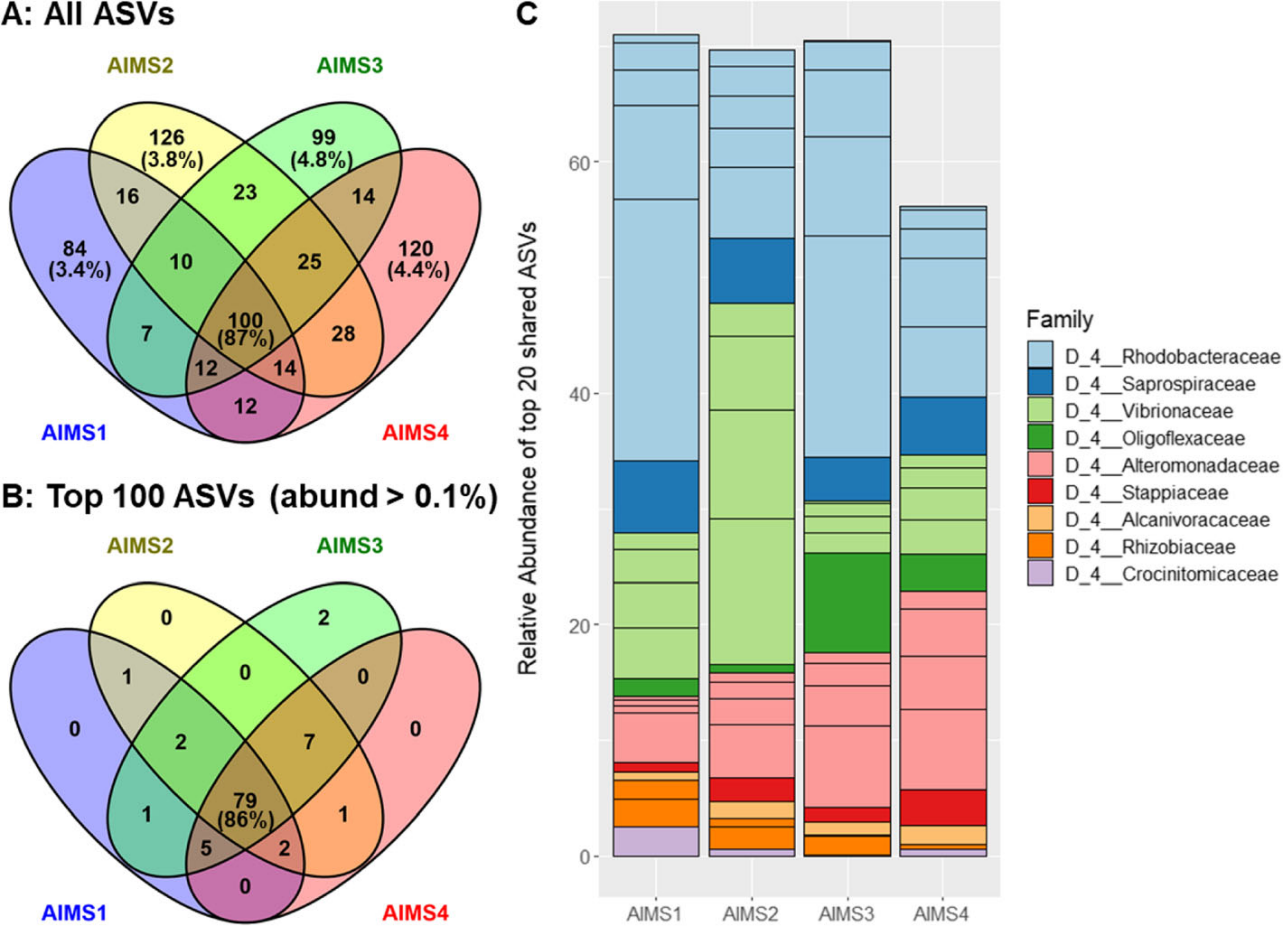
Bacterial ASVs are highly shared in acontia from four genotypes of *E. diaphana*. **a-b**: Venn diagrams of all ASVs (**a**) and top 100 ASVs (**b**; abundance > 0.1% across all samples) present in acontia from genotypes AIMS1–4, based on 16S rRNA gene metabarcoding data. Three independent replicates per genotype were merged for this analysis. Percentages represent the relative abundance of either unique or shared ASVs across each genotype (unique ASVs) or all genotypes (shared ASVs). **c**: Relative abundance of the 20 most abundant ASVs that are shared across all genotypes, grouped by family. Within a given family, different blocks represent distinct ASVs. For each genotype, three independent replicates were merged. See Additional file 4 for full data

Among the 690 ASVs present in the acontia samples, 100 were present in all four genotypes (Fig. 3a). While this only represented 14.5% of all ASVs, these 100 ASVs accounted for 84–89% of all reads (Fig. 3a, Additional file 4A), while unique ASVs represented less than 5% of reads in each genotype (Fig. 3a, Additional file 4B-E). This indicates that the majority of the bacterial taxa are shared across genotypes. In line with this, out of the most abundant ASVs (relative abundance > 0.1% across the dataset, 100 ASVs), 79% were shared by all four genotypes (Fig. 3b), accounting for 83–89% of all reads (Additional file 4F). This shows that abundant ASVs are predominantly shared, attesting to the similarity across acontia from different genotypes. Shared ASVs largely belonged to three families: *Alteromonadaceae, Vibrionaceae*, and *Rhodobacteraceae* (Fig. 3c), with *Vibrio*, *Sulfitobacter*, *Marivita*, *Alteromonas* and *Lewinella* being the most represented genera (Additional files 4A and 4F).

### Acontia bacterial communities significantly differ from those of whole anemones

**Fig. 4:**
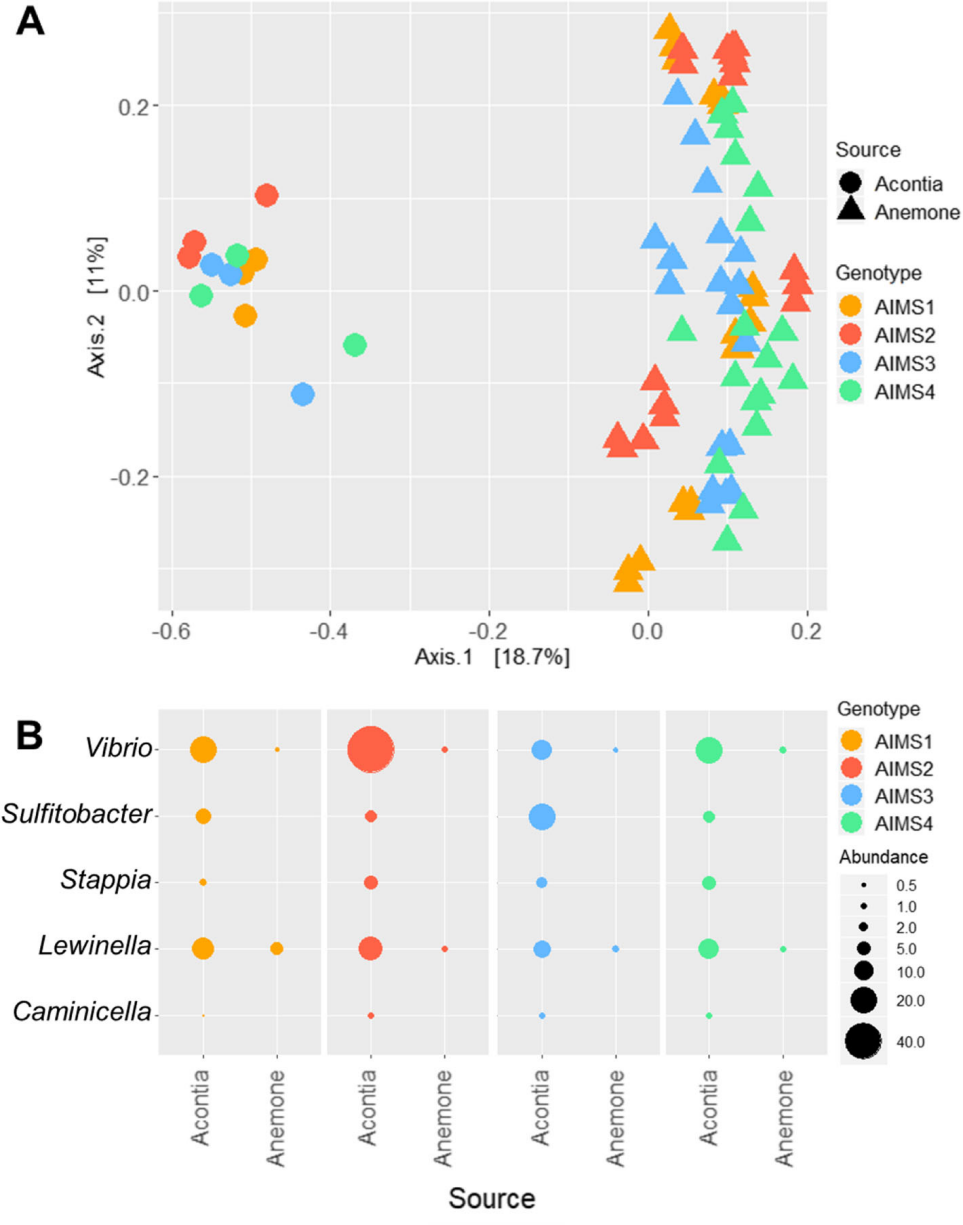
Acontia bacterial communities differ from whole anemone communities. **a**: PCoA visualization of beta-diversity of the bacterial communities in acontia (circles) and whole anemones (triangles) from genotypes AIMS1–4, based on Bray-Curtis dissimilarity matrices on 16S rRNA gene metabarcoding data. Each point is an individual sample. Whole anemone data were used with permission from [47]. For each genotype, bacterial communities were different between acontia and whole anemones (PERMANOVA, 999 permutations). **b**: Relative abundance of five genera that were found to be enriched in acontia (versus whole anemone) based on an indicator value analysis. These five genera were the only ones appearing in acontia from all four genotypes. See Additional file 5 for full data

To investigate which bacteria are enriched in acontia, we compared our dataset with data obtained from whole anemones belonging to the same genotypes and clonal populations, reared in the same conditions, and processed in the same way as in the present study [47]. PCoA visualization using the Bray–Curtis dissimilarity index showed a high degree of clustering by source, with all acontia data points being clearly separated from all anemone data points (Fig. 4a). PERMANOVA testing (999 permutations) by genotype confirmed that bacterial communities are significantly different between acontia and whole anemones, for all genotypes (*F*_AIMS1(1,19)_ = 6.94, *p* = 0.002; *F*_AIMS2(1,19)_ = 5.94, *p* = 0.002; *F*_AIMS3(1,19)_ = 7.45, *p* = 0.002; *F*_AIMS4(1,19)_ = 7.12, *p* = 0.002). An indicator value analysis was performed to identify genera that represent indicators for the acontia (Additional file 5A-D). These genera met a specificity and fidelity threshold of 80%. Five of these genera appeared in all genotypes (Fig. 4b), accounting for 29–56% of all reads in their respective genotype (Additional file 5E). *Vibrio, Sulfitobacter,* and *Lewinella* were the most abundant of those genera. *Marivita* and *Alteromonas* were among the most abundant and shared in acontia from all genotypes (Additional file 4), were also indicator genera for three out of four genotypes (Additional file 5). Indicator genera for acontia had very low abundance in whole anemones, sometimes being absent altogether. This can be explained by three factors: (i) acontia tissues make up a very small portion of a whole anemone; (ii) bacterial load is likely much higher in other parts of the anemone, including the mucus, which is known to harbor high numbers of bacteria in cnidarians [21]; (iii) even though we sequenced negative controls and decontaminated our samples accordingly, low-biomass samples, like acontia, are more prone to reagent and/or environmental contamination. A combination of these factors could have led to acontia-enriched taxa contributing very few reads in whole anemone samples.

## Discussion

Our study describes the microbial communities associated with acontia, defensive tissues of the anemone *E. diaphana*. Despite several reports investigating bacterial communities in anemones [32–36], this is the first time a single compartment has been targeted for community profiling. Symbiodiniaceae were found to be present in acontia, which is consistent with the gastrodermal origin of the mesenteries from which acontia are derived. Symbiodiniaceae were previously identified as *Breviolum minutum* in the four *E. diaphana* genotypes used in this study [37, 48]. While PERMANOVA analysis found host genotype to be a driver of bacterial communities, pairwise comparisons did not detect differences between genotypes, and an analysis of shared ASVs across genotypes showed high consistency between acontia microbiomes across genotypes. This is consistent with a recent paper showing that whole anemone communities from these four same genotypes were not significantly different [35]. Nonetheless, the four genotypes used in this study are reared in very similar environments. It would thus be relevant to sample other genotypes as well as wild populations for comparison of their bacterial community composition with that of the acontia, as whole organism microbiomes have been shown to be more diverse in wild populations [35].

Although acontia bacterial communities were similar across genotypes, they significantly differed with communities associated with whole anemones from the same genotypes and reared under the same conditions. However, acontia and whole anemone data were obtained at different times, and it is therefore possible that sampling time was a factor driving bacterial community differences, as additional bacteria might have been introduced during anemone feeding and/or cleaning. Examination of acontia and anemone microbiome simultaneously, over time, and in different rearing conditions is required to verify whether acontia have a specific microbiome that is distinct from other anemone tissues. It is important to study bacterial communities from different organismal compartments, as function and degree of integration are very likely to be correlated with the location of different bacteria. Here, we identified five genera that might be indicative of the acontia microbiome and exert important roles on the holobiont. These were *Vibrio, Sulfitobacter, Marivita, Alteromonas* and *Lewinella*. Of those, *Vibrio* and *Alteromonas* are often found in coral mucus [49, 50].

Potential functions of these bacteria in the acontia include nutrient cycling, as both *Vibrio* and *Alteromonas* strains have been suggested to play a part in nitrogen and sulfur cycling in corals [51, 52]. *Sulfitobacter* species can also be involved in sulfur cycling [53] and have been shown to provide a marine diatom with a growth-promoting hormone [54]. *Sulfitobacter* and *Marivita* were reported to be enriched in heat-stressed *Porites lutea* corals when compared to controls [55], and thus might have a role in responses to heat stress in anemones. *Vibrio* are well known as pathogens of cnidarians [56–58], including *E. diaphana* where darkening of tissue and tentacle retraction occurs [59, 60], and can be the cause of coral bleaching [61]*.* The anemones in our study were visually healthy, suggesting the observed *Vibrio* strains were not pathogens or were non-virulent. Some *Vibrio* species isolated from anemones and sea cucumbers were shown to exert antibacterial activity [62], supporting this possibility. In addition, it was shown that repetitive exposure of *E. diaphana* to sub-lethal doses of *Vibrio coralliilyticus* could lead to immune priming and improved survival to this pathogen, possibly through the up-regulation of heat shock proteins [60]. Finally, *Vibrio* species can produce tetrodotoxin, a paralyzing neurotoxin, in a wide range of marine organisms [63–65]. Hence, *Vibrio* could be involved in the defensive functions of the acontia. Pure cultures of *Vibrio* were successfully isolated from acontia and should be studied for further functional analyses to test these hypothetical functions.

## Conclusions

In conclusion, we characterized the microbial communities associated with the acontia of *E. diaphana* and hypothesized that some of these bacteria, especially *Vibrio*, may be involved in acontia-related defense processes. Further analyses on cultured isolates, such as genome sequencing or metabolite profiling, may provide insight into the actual function(s) these bacteria have within the acontia. Our findings illustrate the need to study bacterial communities in individual compartments, both in cnidarian models and corals, to fully appreciate and take advantage of the functional diversity of cnidarian-associated microorganisms.

## Material and methods

### Anemone rearing and macroscopic observations

Anemones from four Great Barrier Reef-sourced genotypes were reared as previously described [37]. Briefly, anemones were grown in reverse osmosis water reconstituted Red Sea Salt™ at ~ 34 parts per thousand (ppt), and incubated at 26 °C under lighting of 12–15 μmol photons m^− 2^ s^− 1^ (light emitting diode - LED white light array) on a 12 h:12 h light:dark cycle. Anemones were fed ad libitum twice weekly with freshly hatched *Artemia salina* nauplii. The four genotypes (AIMS 1–4) were previously identified [37] based on sequencing of the 18S rRNA gene, one sequence characterized amplified region (SCAR) marker [66], four *Exaiptasia*-specific gene loci [67], and SNP analysis. All anemones used in this study were reared in the same conditions, and anemones of a given genotype were reared in the same tank. For macroscopic observations, anemones were sampled with sterile pipettes and transferred to 6-well plates in filter sterilised Red Sea Salt Water (fRSS; 34 ppt salinity). Acontia ejection was triggered by poking anemones with tweezers. Images were acquired using a Leica M205FA stereo microscope (Leica Microsystems, Germany).

### Acontia preparation for confocal laser scanning microscopy (CLSM)

Anemones were sampled with sterile pipettes and anesthetized in sterile MgCl_2_ 0.4 M in fRSS. Under a stereomicroscope, acontia ejection was triggered by poking anemones with sterile tweezers, and acontia were subsequently detached from the body with sterile tweezers to ensure no cross contamination from other tissues. Acontia from three anemones per genotype were transferred to paraformaldehyde (PFA) 4% and fixed overnight at 4 °C. Acontia were then transferred to 40 μm mesh size strainers (pluriSelect, Germany) for easy transfer between solutions. Acontia were first rinsed twice in PBS for 5 min to remove any PFA. Acontia were then transferred to a Teflon® printed microscope slide (ProSciTech) and mounted in CitiFluor™ CFM3 mounting medium (Hatfield, PA, USA).

### CLSM

Observations were made on a Nikon C2 CLSM (Nikon, Tokyo, Japan) with the NIS325 Element software. Virtual band mode was used to acquire variable emission bandwidth to tailor acquisition for specific fluorophores. The acontia structure was observed using the transmitted light channel. The chlorophyll of the Symbiodiniaceae cells was excited using the 488 nm laser line, with a 670–720 nm detection range. Nd2 files were processed using ImageJ.

### Cultures of bacteria associated with acontia

Three anemones per genotype were sampled with sterile pipettes, anesthetized in sterile MgCl_2_ 0.4 M in fRSS, and acontia were sampled as described above. Acontia were deposited in 40 μm mesh size strainers (pluriSelect) and briefly rinsed with fRSS, 80% ethanol, and fRSS again, to remove any debris outside the acontia. Acontia were then homogenized in a glass homogenizer in 500 μL fRSS. Homogenized solution from each genotype was diluted by 10, 100, and 1000. 50 μL of each dilution were spread on Petri plates of BD Difco™ Marine Agar 2216 (MA) and Oxoid Reasoner’s 2A (R2A) agar prepared with fRSS. Plates were incubated at 26 °C for 7 days to facilitate bacterial colony growth. The morphology of bacterial colonies including form, elevation, margin, surface, texture, colour, and opacity were recorded and representatives of all morphologies from plates with less than 300 colonies per plate were subcultured by the 16-streak method to attain culture purity. Pure cultures were stored in sterile 40% glycerol at − 80 °C.

### 16S rRNA gene sequencing from cultured bacteria

Individual pure freshly grown bacterial colonies were suspended in 20 μL sterile Milli-Q® water, incubated for 10 min at 95 °C then used as templates in colony PCRs. PCR amplification of the bacterial 16S rRNA gene was with primers 27f and 1492r [68] in reactions containing 1 μL template DNA, 0.5 μL of each primer (10 μM, final concentration: 0.33 μM), 7.5 μL of 2x MyTaq HS Red Mix (Bioline) and 5.5 μL of sterile PCR-grade water. The amplification cycle was: 5 min at 94 °C; 30 cycles of 60 s at 94 °C, 45 s at 50 °C, and 90 s at 72 °C; 10 min at 72 °C; with a final holding temperature of 4 °C. The PCR products were Sanger sequenced with primer 1492r at Macrogen (South Korea). Raw sequences were trimmed and proofread in Mega 7.0 (https://www.megasoftware.net/home).

### Phylogenetic tree construction

Each individual 16S rRNA gene sequence was aligned using SILVA SINA alignment tool and the SILVA reference alignment [69]. The SILVA reference alignment searched the related sequences (two nearest neighbors) to 95% min identity of the 16S rRNA gene sequences from this study. The full alignment was stripped of columns containing 99% or more gaps, generating a final alignment containing 243 taxa (150 from this study) and 1594 nucleotides. A maximum likelihood tree was inferred using RAxML-HPC BlackBox [70] as implemented on the CIPRES [71] web server under the GTRCAT evolutionary model. The RAxML inference included the calculation of 360 bootstrap iterations, with 100 randomly sampled to determine support values.

### Sampling and DNA extraction for 16S rRNA gene metabarcoding

Five anemones per genotype were sampled with sterile pipettes, anesthetized in sterile MgCl2 0.4 M in fRSS, and acontia were sampled as described above. Acontia were deposited in 40 μm mesh size strainers (pluriSelect) and briefly rinsed with fRSS, ethanol 80%, and fRSS again, to remove any debris outside the acontia. Acontia were then homogenized in a glass homogenizer in 500 μL fRSS, snap-frozen and kept at − 80 °C until DNA extraction. DNA extraction were performed according to the Wilson’s method [72] with modifications as previously described [48]. One blank DNA extraction was conducted as a negative control.

### 16S rRNA gene PCR amplification, library preparation and sequencing

Hypervariable regions V5-V6 of the 16S rRNA genes were amplified using the primer set 784F (5′ GTGACCTATGAACTCAGGAGTCAGGATTAGATACCCTGGTA 3′) and 1061R (5′ CTGAGACTTGCACATCGCAGCCRRCACGAGCTGACGAC 3′) [73]. Illumina MiSeq adapters were attached to the primers and are shown as underlined. Bacterial 16S rRNA gene by PCR on a SimpliAmp Thermal Cycler (Applied Biosystems, ThermoFisher Scientific). Each reaction contained 1 μL of DNA template, 1 μL of forward primer (10 μM stock), 1 μL of reverse primer (10 μM stock), 7.5 μL of MyTaq HSRed MasterMix (BioLine), and 4.5 μL of nuclease-free water (Thermofisher), with a total volume of 15 μL per reaction. Three triplicate PCRs were conducted for each sample and one no template PCR was conducted as a negative control. PCR conditions for the 16S rRNA genes were as follows: initial denaturation at 95 °C for 3 min, then 18 cycles of: denaturation at 95 °C for 15 s, annealing at 60 °C for 30 s, and extension at 72 °C for 30 s; with a final extension at 72 °C for 7 min. Samples were then held at 4 °C. Following PCR, triplicates were pooled, resulting in 45 μL per sample.

Metabarcoding library preparation and sequencing was performed at the Walter and Eliza Hall Institute (WEHI) (Melbourne, Australia) on one MiSeq V3 system (Illumina) with 2x300bp paired-end reads. Library preparation involved addition of 20 μL of next-generation sequencing magnetic beads to 20 μL of PCR product (1:1), for clean-up to ensure high quality down-stream sequencing. Beads were washed twice with 70% ethanol, and DNA was resuspended with 40 μL of nuclease-free water. 10 μL of cleaned-up PCR products were combined with 10 μL 2x Taq MasterMix (M0270L, New England BioLabs) and 0.25 μM of forward and reverse indexing primers. The second PCR conditions were as follows: initial denaturation at 95 °C for 3 min, 24 cycles of: 95 °C for 15 s, 60 °C for 30 s, and 72 °C for 30 s; followed by a final extension at 72 °C for 7 min. Product size and specificity of two replicates of representative 16S rRNA gene amplifications were assessed using the TapeStation (2200 TapeStation, Agilent Technologies). A final bead clean-up was performed on a pool of 5 μL from each well per plate. Pooled libraries were checked for quality control, size determination, quantity and purity of each sample, to inform pool normalisation by using the TapeStation (2200 TapeStation, Agilent Technologies).

### Bacterial 16S rRNA gene analysis

QIIME2 v 2019.4.0 [74] was used for processing 16S rRNA gene sequences. The plugin demux [74] was used to create an interactive plot to visualise the data and assess the quality, for demultiplexing and quality filtering of raw sequences. The plugin cutadapt [75] was used to remove the primers and Illumina MiSeq adapters. Plugin DADA2 [76] was used for denoising and chimera checking, trimming, dereplication, generation of a feature table, joining of paired-end reads, and correcting sequencing errors and removing low quality reads (Q-score < 30). Summary statistics were obtained using the feature-table to ensure processing was successful. Taxonomy was assigned by training a naive Bayes classifier with the feature-classifier plugin [74], based on a 99% similarity to the V5-V6 region of the 16S rRNA gene in the SILVA 132 database to match the 784F/1061R primer pair used [77]. Alignment [78] and phylogeny [79] packages enabled the production of a phylogenetic tree for later analyses in R Studio. Metadata file, phylogenetic tree, and tables with Amplicon Sequence Variant (ASV) taxonomic classifications and counts were imported into R for statistical analyses.

### Statistical analyses in R studio

Statistical analyses and graphs were performed using R version 3.5.0 [80], and the packages phyloseq [81], vegan [82], RVAideMemoire [83], ggplot2 [84], tidyverse [85], indicspecies [86]. Statistical tests were considered significant at α = 0.05, unless otherwise stated. Metadata file, taxonomy table, phylogenetic tree and ASV table were imported into R and mitochondria and chloroplast sequences were removed. Contaminants ASVs were identified manually based on their abundance in negative controls: any ASVs that was five times more abundant in either the extraction blank or the no template PCR, and that represented at least 50 reads in other samples was considered a contaminant and removed from the dataset. 22 putative contaminant ASVs were identified, constituting 7.3% relative abundance of the bacterial communities in acontia samples (Additional file 6).

Alpha diversity metrics (observed ASVs, Simpson index, Shannon index) were calculated after rarefying the samples to 12,460 reads per sample. Alpha diversity data were then analyzed for overall differences using ANOVA, after checking that data had a normal distribution and homogenous variances using Shapiro and Levene tests, respectively.

Differences in community composition (β-diversity, [87]) were computed using Bray–Curtis dissimilarity matrices and tested via permutational multivariate analysis of variance (PERMANOVA, [88]). Variation in community composition among samples was visualized with PCoA. A test for multivariate homogeneity of group dispersions (PERMDISP, [89]) was used to check for homogeneity of variances and pairwise comparisons were performed between groups using the Benjamin and Hochberg [90] correction for multiple testing. Venn diagrams were constructed using Venny 2.1.0 [91].

Whole anemone data used for indicator value analysis was previously published (PRJNA576556 [47];) These data were obtained from whole anemones deriving from the same clonal populations used for acontia sampling. Anemones from both studies were derived from the same clonal populations, maintained in the same lab and in the same rearing conditions. Sampling, DNA extractions, and 16S rRNA gene metabarcoding was conducted with the same protocols and at the same facility. Data analysis was performed through the same pipelines. The indicator value analysis [86] was applied to detect genera that were significantly associated with acontia (versus whole anemones) when both specificity and fidelity had probabilities > 80%. Comparisons (PCoA, PERMANOVA, indicator species) were performed at the genus level to avoid ASV-level differences that could result from data processing differences.

## Supplementary Information

**Additional file 1.** Visualization of acontia (grey, transmitted light) in fluorescence microscopy highlighting the presence of Symbiodiniaceae (blue) in AIMS2 (A), AIMS3 (B), and AIMS4 (C) genotypes. Note the presence of a tentacle in A, highlighting the much higher Symbiodiniaceae density in tentacles than in acontia. Scale bars: 100 μm (left column); 20 μm (right column). Blue and grey are not the real colors.

**Additional file 2 **Database of bacteria isolated from acontia from *E. diaphana*. Is reported for each isolate: source genotype, partial 16S rRNA sequence, morphology, closest BLAST result and full taxonomy. When there were several BLAST results with identical scores, the species column was left on “sp.”.

**Additional file 3.** Maximum likelihood phylogeny of the 16S rRNA gene placing the acontia-associated cultured bacterial symbionts within the broader phylogeny of the bacteria. The final alignment contained 243 sequences, 150 from this study, and was generated using the SILVA SINA alignment tool and the SILVA reference alignment. The tree was constructed using RAxML-HPC under the GTRCAT model of evolution. See Table S1 for a full description including colony morphology, 16S rRNA gene sequences, and taxonomic affiliations of the 150 isolates cultured from acontia.

**Additional file 4.** Abundance of ASVs shared across all four genotypes AIMS1–4 (A, F) or unique to a given genotype (B-E). Only ASVs with a mean abundance > 0.1% are shown in F.

**Additional file 5.** Abundance of genera specific to acontia (versus whole anemones) based on an indicator value analysis (A-D). Five genera were present in all genotypes, and their abundance across all genotypes is presented in E.

**Additional file 6.** List of contaminants identified in the 16S rRNA metabarcoding data.

## Data Availability

Genbank accession numbers for 16S rRNA sequences of cultured bacteria are MT840368-MT840517 (see Additional file 2). Raw Illumina MiSeq data from acontia are available under NCBI BioProject ID PRJNA650220. Raw Illumina MiSeq data from whole anemones [47] are available under NCBI BioProject ID PRJNA576556.

## Abbreviations

AIMS: Australian Institute of Marine Science
ASV: Amplicon sequence variant
CLSM: Confocal laser scanning microscopy
fRSS: Filtered Red Sea salt water
PCoA: Principal component analysis
R2A: Reasoner’s 2A agar
