# Supplementary figures and images for: Microbiome characterization of defensive tissues in the model anemone Exaiptasia diaphana
Source: BMC Microbiol. 2021 May 21;21:152. doi: 10.1186/s12866-021-02211-4 (PMC8140459; doi:10.1186/s12866-021-02211-4)

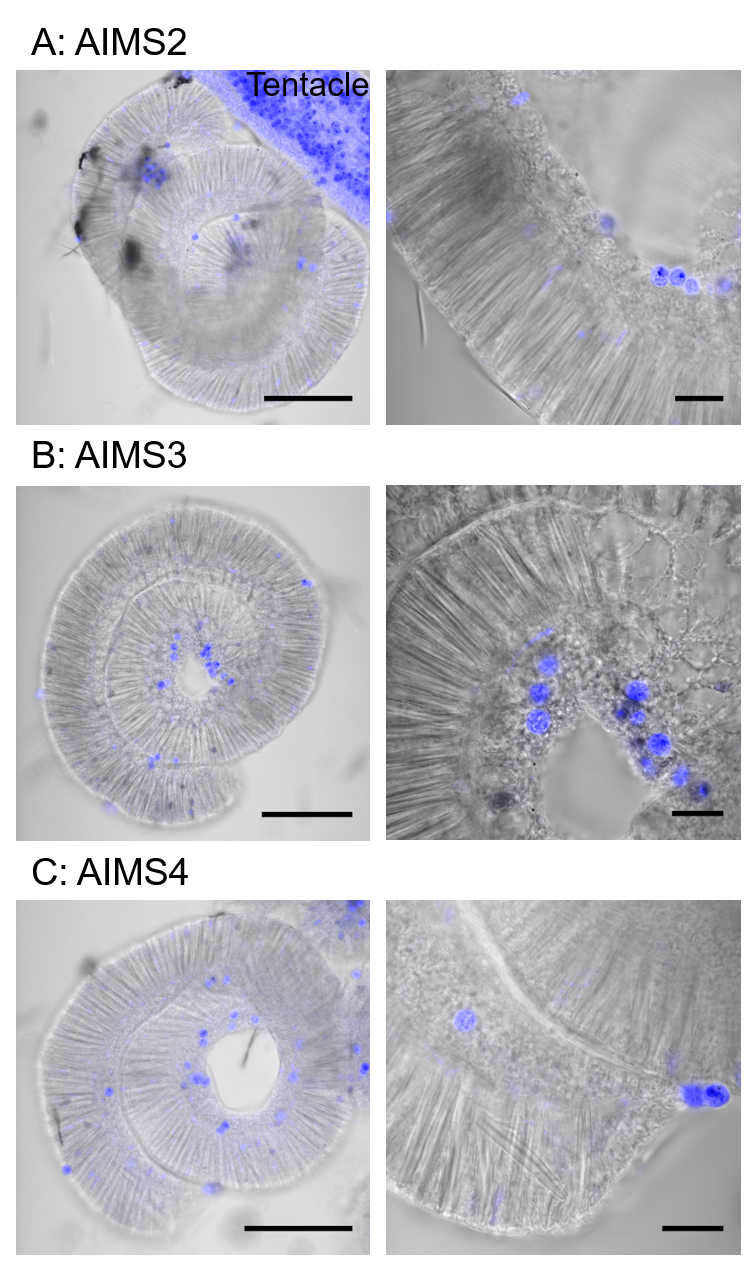

Supplement: Supplementary file 1 — Additional file 1. Visualization of acontia (grey, transmitted light) in fluorescence microscopy highlighting the presence of Symbiodiniaceae (blue) in AIMS2 (A), AIMS3 (B), and AIMS4 (C) genotypes. Note the presence of a tentacle in A, highlighting the much higher Symbiodiniaceae density in tentacles than in acontia. Scale bars: 100 μm (left column); 20 μm (right column). Blue and grey are not the real colors. [file 12866_2021_2211_MOESM1_ESM.tif]

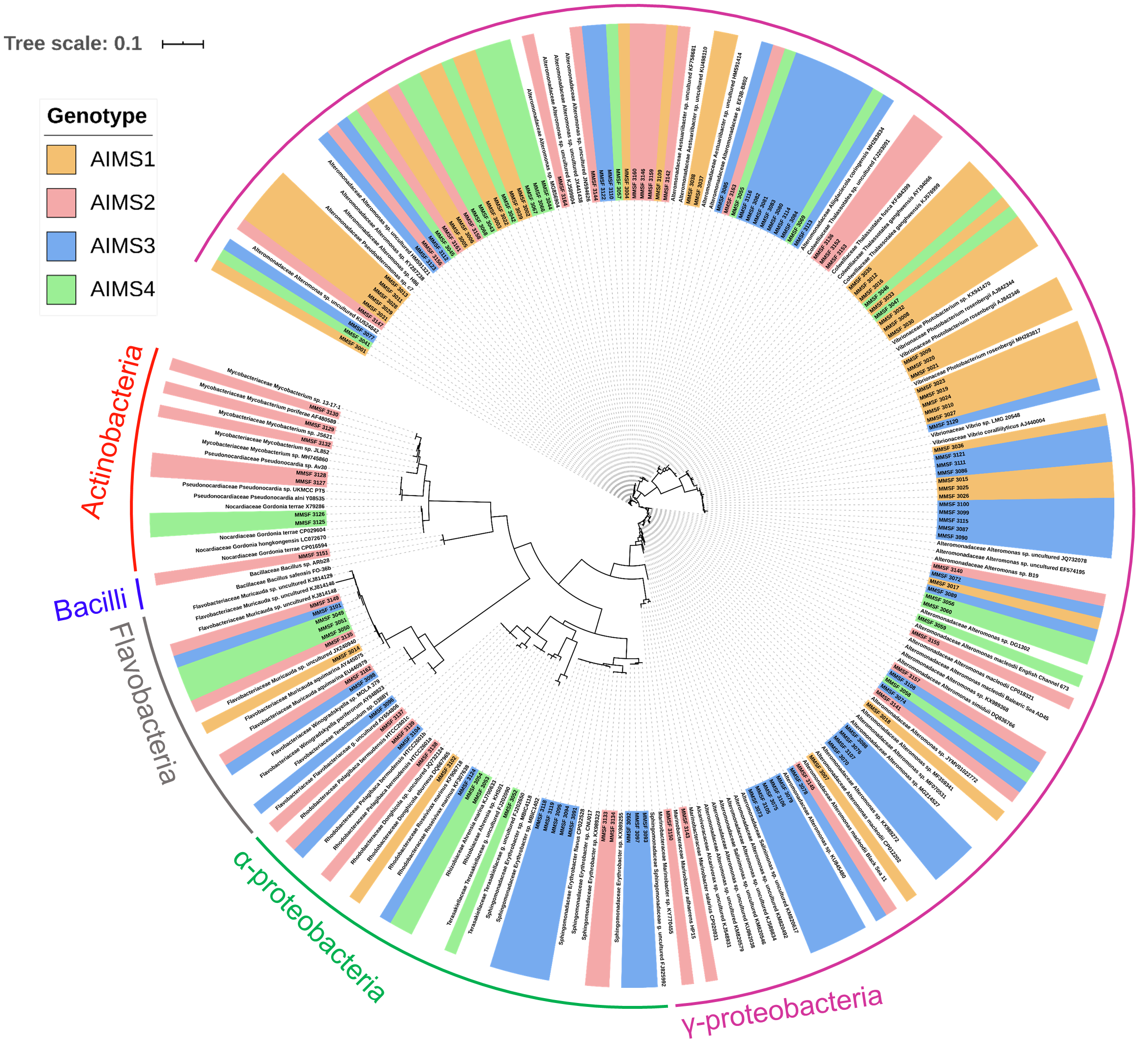

Supplement: Supplementary file 3 — Additional file 3. Maximum likelihood phylogeny of the 16S rRNA gene placing the acontia-associated cultured bacterial symbionts within the broader phylogeny of the bacteria. The final alignment contained 243 sequences, 150 from this study, and was generated using the SILVA SINA alignment tool and the SILVA reference alignment. The tree was constructed using RAxML-HPC under the GTRCAT model of evolution. See Table S1 for a full description including colony morphology, 16S rRNA gene sequences, and taxonomic affiliations of the 150 isolates cultured from acontia. [file 12866_2021_2211_MOESM3_ESM.tif]
